# Getting to the heart of a good fossil

**DOI:** 10.7554/eLife.16207

**Published:** 2016-04-19

**Authors:** John A Long

**Affiliations:** School of Biological Sciences, Flinders University, Adelaide, Australiajohn.long@flinders.edu.au

**Keywords:** cardiac, development, evolution, fishes, Cretaceous, fossils, None

## Abstract

The discovery of perfectly preserved 113-119 million year old fossilised hearts in a Brazilian fish *Rhacolepis* has significant implications for palaeontology and comparative anatomy.

**Related research article** Maldanis L, Carvalho M, Almeida MR, Freitas FI, Andrade JAFG, Nunes RS, Rochitte CE, Poppi RJ, Freitas RO, Rodrigues F, Siljeström S, Lima FA, Galante D, Carvalho IS, Perez CA, de Carvalho MR, Bettini J, Fernandez V, Xavier-Neto J. 2016. Heart fossilization is possible and informs the evolution of cardiac outflow tract in vertebrates. *eLife* **5**:e14698. doi: 10.7554/eLife.14698

**Image** A fossilised fish heart with the cardiac chambers highlighted in blue

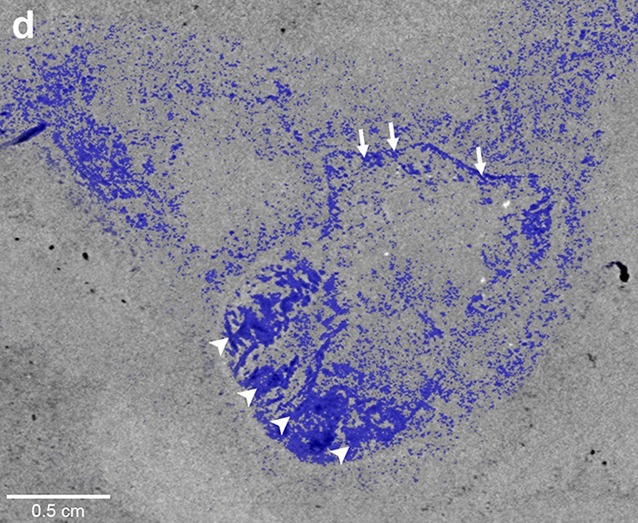

Like the famed Tin Man in *The Wizard of Oz*, if we palaeontologists ‘only had a heart’ we could do a lot more good science interpreting the significance of fossilised remains. For centuries, the fossil remains of ancient back-boned animals have been studied primarily from bones (skeletons) or traces (fossilised footprints). The possibility of finding preserved fossilised soft tissues was widely thought to be impossible as organic soft materials routinely decay in the process of burial and fossilisation.

However, certain fossil deposits, called *Konservat Lagerstätten*, are the result of rapid burial and special chemical conditions involving low-oxygen environments. These deposits can preserve or mineralogically “ghost” a range of soft tissues, even complete organs, in the fossilised organism.

Scientists have long known about the famous Burgess Shale fossils of soft-bodied worms and other invertebrate creatures buried by rapid mud slides around 508 million years ago. However, well-preserved fishes from the 113–119 million year old Santana Formation of Brazil were amongst the first vertebrate fossils to show evidence of preserved soft tissues. These include parts of the glandular stomachs and bands of muscles, with the original tissue having been mostly replaced by chemical processes (Martill, 1988; Wilby and Martill, 1992).

Finding whole preserved internal organs was a bit of a Holy Grail for palaeontology. Such discoveries can contribute a wealth of new anatomical information that is essential for understanding evolutionary patterns. Therefore the finding of a complete, well-preserved fossilised heart in an almost 120 million year old fish – now reported in eLife – is a major breakthrough for José Xavier-Neto of the Brazilian Biosciences National Laboratory, Vincent Fernandez of the European Synchotron Radiation Facility and colleagues from across Brazil and Sweden, including Lara Maldanis and Murilo Carvalho as joint first authors (Maldanis et al., 2016).

The new discovery was made by using synchrotron X-ray tomography to image fossils of the extinct fish *Rhacolepis* that were still entombed within limestone concretions. This technique images the fossil in thin sections; these images can then be processed to render the heart slice by slice and digitally restore the features of the organ. This method has been widely applied in palaeontology for the past decade or so to reveal many intricate soft tissue structures in fossils. These include the preserved brain of a 300 million year old fish from North America (Pradel et al., 2010) and a collection of superbly preserved soft tissues in 380 million year old fishes in Western Australia, such as an embryo with a mineralised umbilical cord (Long et al., 2008), nerve axial plate cells and muscle bundles (Trinajstic et al., 2007; 2013).

The *Rhacolepis* heart is the first example of a complete fossilised soft organ in an extinct fish, and the first fossilised heart in any fossil vertebrate (news reports from 2000 describing a dinosaur with a heart preserved have been recently disproven; Cleland et al., 2011). The heart shows excellent detail of the conus arteriosus, the conical extension of the ventricle that in certain fishes helps regulate blood outflow via the valves in the conus. It also shows the pattern of five rows of valves inside it. The anatomical interpretation of Maldanis, Carvalho et al. is further reinforced by detailed comparisons with dissected hearts from closely-related living fishes called tarpons, which show similar structures in the same relative positions.

The discovery of the fossilised *Rhacolepis* heart is significant because the range of valve patterns in early ray-finned fish hearts is strikingly diverse. Some, like the very primitive (“basal”) ray-finned fish *Polypterus* (the African reedfish), have nine rows of valves, whereas most of the modern group of ray-fins, the teleosts, have just a single outflow valve in the heart.

In between these two groups of fishes we can now enter *Rhacolepis*, a fish belonging to an extinct group that has a basal position amongst the teleosts (Arratia, 2010; Figure 1). Thus the five rows of valves shown by the fossil seems to represent a good intermediate condition between the most primitive pattern and the most advanced type. However, as we well know, in biology simple patterns often hold more complex underlying meanings. For example, the valve pattern within the conus arteriosus has simplified independently in sturgeons and bowfins. There is also evidence for an independent increase in the numbers of valves in some basal ray-finned fishes (Lepisosteiformes, Polypteriformes), so interpreting evolutionary patterns from one data point is always risky business. Maldanis, Carvalho et al. claim that the *Rhacolepis* heart supports a possible case of gradual speciation (as opposed to drastic anatomical changes from one generation to the next). With only one data point, this hypothesis cannot be tested further.

**Figure 1.**
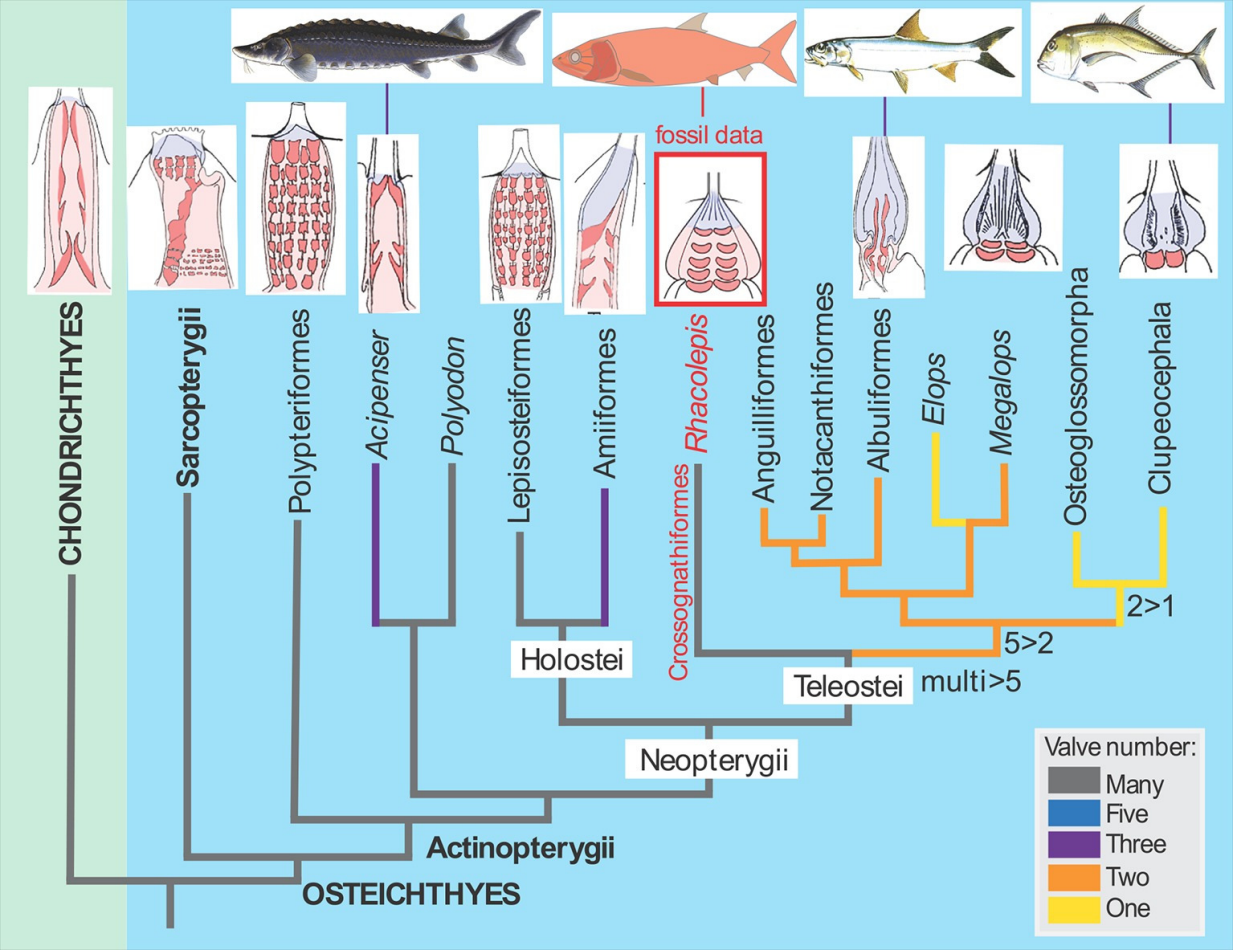
The fossil heart data from *Rhacolepis* shows an intermediate condition between the many-valved types seen in basal ray-finned fishes (actinopterygians) like Polypteriformes (the order that includes the African reedfish) and the single-valved hearts in modern teleosts. Images of the hearts showing valves (red) are oriented with the top of the image pointing towards the head of the fish (taken from Maldanis et al., 2016).

Nonetheless, for the first time we actually do have a data point to study the detailed anatomy of a fossilised heart in an extinct group of fishes. The find demonstrates the immense potential for more discoveries of this nature, enabling more discussion of the comparative anatomy of soft organs in extinct animals. With more highly detailed discoveries like this one, I confidently predict we will one day be able to really get to the heart of resolving the mysteries of early vertebrate evolution. That day is not far away.
